# The causal relationship between rheumatoid arthritis and bronchiectasis: a bidirectional Mendelian randomization study

**DOI:** 10.3389/fmed.2024.1403851

**Published:** 2024-06-20

**Authors:** Yuanyuan Li, Weina Wang, Dengfeng Zhou, Lili Li

**Affiliations:** ^1^Department of Respiratory and Critical Care Medicine, Wuhan Fourth Hospital, Wuhan, Hubei, China; ^2^Department of Gastroenterology, Wuhan Fourth Hospital, Wuhan, Hubei, China

**Keywords:** rheumatoid arthritis, bronchiectasis, Mendelian randomization, causal association, GWAS

## Abstract

**Background:**

Several observational studies suggested an association between rheumatoid arthritis (RA) and bronchiectasis. Nevertheless, the presence of a causal relationship between these conditions is yet to be determined. This study aimed to investigate whether genetically predicted RA is associated with the risk of bronchiectasis and vice versa.

**Methods:**

We obtained RA genome-wide association study (GWAS) data from FinnGen consortium, and bronchiectasis GWAS data from IEU Open GWAS project. Univariate Mendelian randomization (MR) analysis was performed using inverse variance weighted (IVW) estimation as the main method. Furthermore, bidirectional and replication MR analysis, multivariate MR (MVMR), Mediation analysis, and sensitivity analyses were conducted to validate the findings.

**Results:**

In the UVMR analysis, the IVW results revealed that RA had an increased risk of bronchiectasis (OR = 1.18, 95% CI = 1.10–1.27; *p* = 2.34 × 10^−6^). In the reverse MR analysis, no evidence of a causal effect of bronchiectasis on the risk of RA was detected. Conversely, in the replication MR analysis, RA remained associated with an increased risk of bronchiectasis. Estimates remained consistent in MVMR analyses after adjusting for the prescription of non-steroidal anti-inflammatory drugs (NSAIDs) and glucocorticoids. Immunosuppressants were found to mediate 58% of the effect of the RA on bronchiectasis. Sensitivity analyses confirmed the stability of these associations.

**Conclusion:**

This study demonstrated a positive causal relationship between RA and an increased risk of bronchiectasis, offering insights for the early prevention of bronchiectasis in RA patients and shedding new light on the potential role of immunosuppressants as mediators in promoting the effects of RA on bronchiectasis.

## Introduction

Rheumatoid arthritis (RA) is a prevalent autoimmune and chronic inflammatory disease (1), affecting approximately 0.3 to 1% of the population in developed countries (2). RA is a chronic autoimmune disorder that primarily affects the joints but is also associated with several well-documented extra-articular manifestations, including ocular, cutaneous, gastrointestinal, cardiac, and pulmonary involvements (3). RA has the potential to impact various anatomical regions of the chest, encompassing the lung parenchyma, both large and small airways, the pleura, and, less commonly, the pulmonary vessels (4, 5). Bronchiectasis is a recognized extra-articular manifestation of RA, characterized by irreversible damage, widening, and thickening of the bronchi, which results in excessive mucus secretion (6, 7). The presence of both bronchiectasis and RA is associated with a diminished quality of life and an elevated risk of infection for patients with RA (8). Additionally, the coexistence of bronchiectasis and RA markedly impacts patients’ health and life expectancy, elevating their risk of mortality. Patients with both conditions face a mortality risk that is 7.3 times higher than that of the general population, five times greater than that of those with RA alone, and 2.4 times higher than that of those with bronchiectasis alone (9). Accordingly, understanding the interplay between bronchiectasis and RA and the underlying mechanisms that guide their relationship is crucial.

The coexistence of bronchiectasis in patients with RA has been recognized for several decades. The link between RA and bronchiectasis was initially delineated in 1955 (10). Since then, many observation studies have shown a close relationship between RA and bronchiectasis. A recent meta-analysis including 23 observational studies revealed that the prevalence of bronchiectasis in RA is 21.1% (95% CI: 15.0–28.9%), as estimated using a random-effects model. The pooled prevalence for clinically defined bronchiectasis was 2.69% (95% CI: 1.63–4.42), whereas that for radiologically identified disease was 24.9% (95% CI: 19.21–31.67). However, the majority of studies have been cross-sectional, enrolling a limited number of patients and lacking control groups (11). Consequently, the direction of the association between these two diseases remains ambiguous. Furthermore, these observational studies are prone to confounding factors and the possibility of reverse causation, leaving it uncertain whether having RA elevates the risk of bronchiectasis.

Mendelian randomization (MR) has garnered significant interest as a tool for elucidating causal relationships between risk factors and disease outcomes. MR capitalizes on random genetic variants, which arise from meiosis, to serve as instrumental variables (IVs) for examining the link between environmental exposures and disease development. The random assignment of genetic variants at conception, preceding the onset of disease, allows MR analyses to mitigate confounding and identify the causal determinants of specific outcomes (12–14). Recent MR studies have confirmed the causal association of RA with pre-eclampsia (15), hypothyroidism (16), lung cancer, etc. (17). Therefore, this study collected published data and employed bidirectional MR analysis to ascertain whether a bidirectional causal relationship exists between RA and bronchiectasis.

## Materials and methods

### Study design

**Figure 1:**
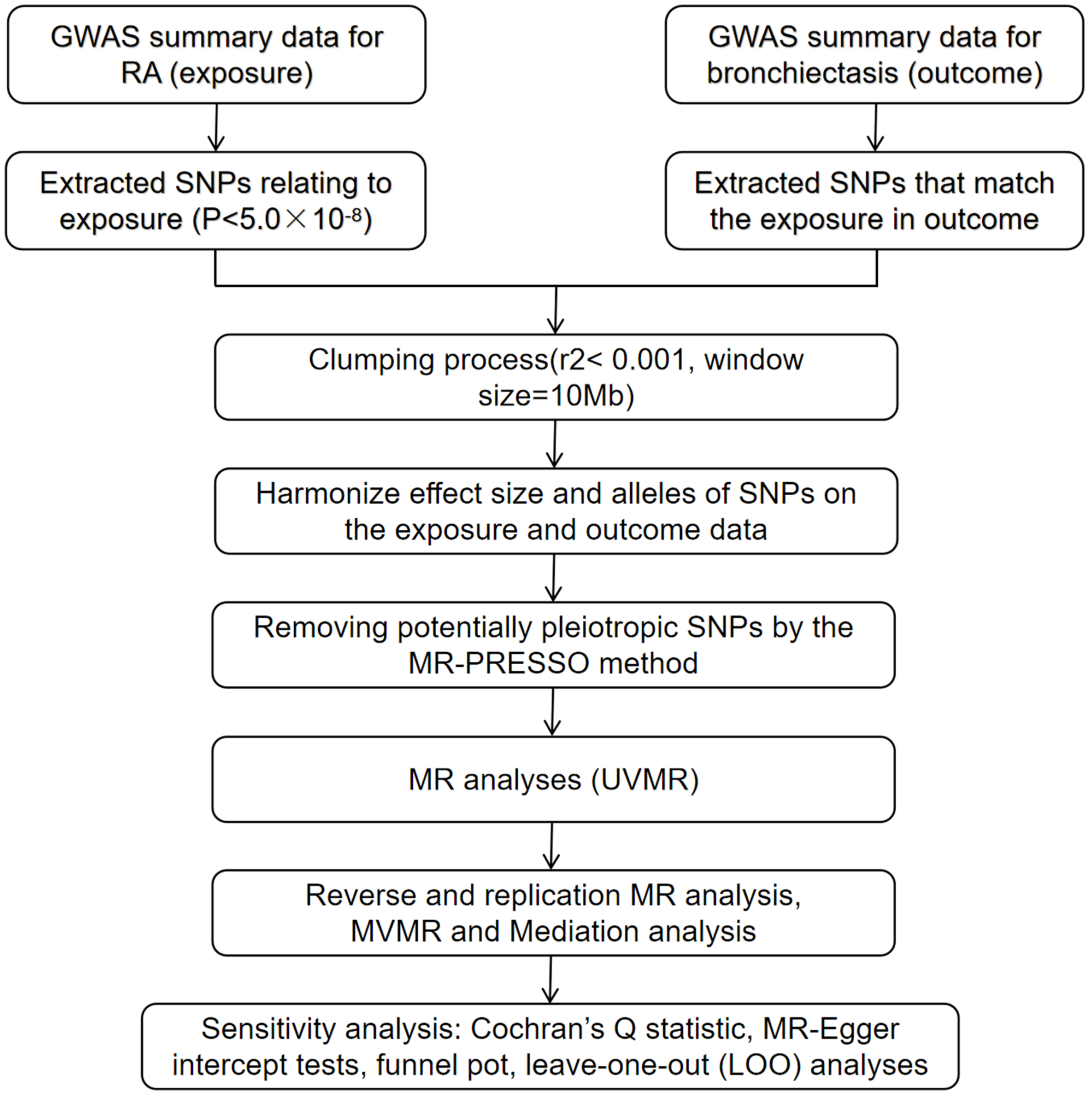
Assumptions and study design of the bidirectional MR study of the associations between RA and bronchiectasis. RA, rheumatoid arthritis; MR, Mendelian randomization; SNPs, single-nucleotide polymorphisms.

This study was conducted following the Strengthening the Reporting of Observational Studies in Epidemiology using MR (STROBE-MR) guidance (Supplementary Table S1) (18). This study did not necessitate a separate ethical review submission. Employing summary statistics from genome-wide association studies (GWAS), we performed two-sample MR analyses to estimate the causal impact of RA on bronchiectasis within European populations. IVs were selected based on three crucial criteria: they should exhibit a strong association with the exposure of interest, be independent of unmeasured confounders, and affect the results exclusively through their relationship with the exposure (19). In this MR study, RA was considered the exposure, while bronchiectasis was the outcome of interest. We initially conducted univariable MR (UVMR) to investigate the potential causal relationship between RA and bronchiectasis. Subsequently, we employed reverse and replication MR analyses to corroborate this relationship. Furthermore, we performed multivariable MR (MVMR) to evaluate the direct effect of RA on bronchiectasis, accounting for potential confounders such as the prescription of non-steroidal anti-inflammatory drugs (NSAIDs), glucocorticoids and immunosuppressants. A concise illustration of the MR study design is provided in Figure 1.

### Data sources

SNPs associated with RA in European populations were identified from a comprehensive GWAS, which included 6,236 RA cases and 147,221 controls from the FinnGen consortium. For bronchiectasis, GWAS data from a European population were sourced from the IEU Open GWAS project, consisting of 2,888 cases and 440,263 controls (20).

### Selection of IVs

In this MR study, single nucleotide polymorphisms (SNPs) were considered significant at the genome-wide level (*p* < 5.0 × 10^−8^). SNPs that exhibited no linkage disequilibrium (LD) with other SNPs (*r*^2^ < 0.001 within a 10 Mb clustering window) were selected as IVs. The F-statistic was computed to evaluate the extent of genetic variation, and SNPs with an F-statistic below 10 were excluded, as they suggested insufficient strength (21). For the missing SNPs in the resulting GWAS dataset, proxies were identified at the cut-off of *R*^2^ > 0.8.^1^ SNPs lacking suitable proxies were removed from the analysis. Harmonization processes were conducted to eliminate ambiguous and palindromic SNPs, as well as those exhibiting high correlation with known results (*p* < 5.0 × 10^−8^). Prior to each MR analysis, MR-pleiotropy residual sum and outlier (MR-PRESSO) tests were conducted. These tests are intended to identify horizontal pleiotropy in fewer than 50% of instruments, allowing for the detection and exclusion of potential outliers (22).

### UVMR

We carried out an MR analysis to estimate the causal effect of RA on bronchiectasis risk. When a single SNP served as the IV, the Wald ratio was employed. In instances with more than two SNPs, the inverse variance weighted (IVW) estimation method was adopted as the primary analysis approach, which involved combining the individual SNPs with their corresponding Wald ratios to derive a pooled estimate of causality. This technique accommodates overdispersion (23). Additionally, complementary MR analyses, including MR-Egger regression and weighted median, were conducted to enhance the reliability of the estimates provided by the IVW method across a broader spectrum of scenarios. The MR-Egger regression is particularly adept at quantifying multinomial and substantial heterogeneity in exposure imbalances; however, it requires a larger sample size to detect comparable levels of variability in under-exposure when compared to alternative methods (24). When at least half of the weighted variance is due to horizontal pleiotropy and is valid, the weighted median method yields consistent effect estimates (25). The *P* < 0.05 (two-tailed) was statistically significant.

### Bidirectional and replication MR analysis

To evaluate the bi-directional causation effects between RA and bronchiectasis, we used bronchiectasis as “exposure” and RA as “outcome.” In addition, to prove the reliability of our findings, we replicated the IVW analysis using another independent RA GWAS data from the IEU Open GWAS project, including 14,361 RA patients, 43,923 controls, as well as 13,108,512SNPs (26). Genetic variants associated with bronchiectasis from the FinnGen consortium, which contained 1,107 bronchiectasis patients, 186,723 controls, as well as 16,380,375 SNPs.

Similarly, we also validated our findings in an East Asian population. SNPs associated with RA were identified from a large GWAS involving 4,199 cases of East Asian descent and 208,254 controls. We further retrieved genetic data related to bronchiectasis in East Asian populations from the IEU Open GWAS project, which included 241 cases and 161,803 controls. The method for performing MR analyses in reverse and replication MR is the same as previously described.

### MVMR

MVMR, using the MVMR-IVW method, was conducted to minimize the correlated pleiotropy introduced by potential confounding factors, including the prescription of NSAIDs, glucocorticoids, and immunosuppressants (27). In MVMR, we obtained summary data for NSAIDs (*n* = 164,520), glucocorticoids (*n* = 205,700), and immunosuppressants (*n* = 272,602) (20) from the IEU Open GWAS project.

### Mediation analysis

We further conducted a mediation analysis using the two-step MR approach to investigate whether the causal effect of RA on bronchiectasis is mediated by the use of immunosuppressants. In the initial step, we determined the causal effect of RA on immunosuppressants (β1). In the second step, we estimated the causal effect of immunosuppressants on the development of bronchiectasis (β2). The mediation effect is calculated as β1*β2. Thus, the proportion mediated could be calculated as β1*β2/the overall effect (β).

### Sensitivity analysis

To further assess the stability of the results, we also conducted Cochran’s Q statistic, MR-Egger intercept, funnel plot, and leave-one-out (LOO) analyses to detect the presence of heterogeneity and pleiotropy. Cochran’s *Q*-test (*p* < 0.05 indicates heterogeneity) and I^2^ statistic (*I*^2^-value > 50% indicates heterogeneity) were utilized to evaluate the heterogeneity among SNPs in the IVW estimates. A *p*-value of <0.05 was deemed indicative of significant heterogeneity, prompting the use of a random-effects model for subsequent analyses. The intercept term in the MR-Egger regression was employed to assess the presence of pleiotropy (25, 28–30). All of the analyses were run by using the R package TwoSampleMR (version 0.5.6) in R (version 4.1.1).

## Results

### UVMR

**Figure 2:**
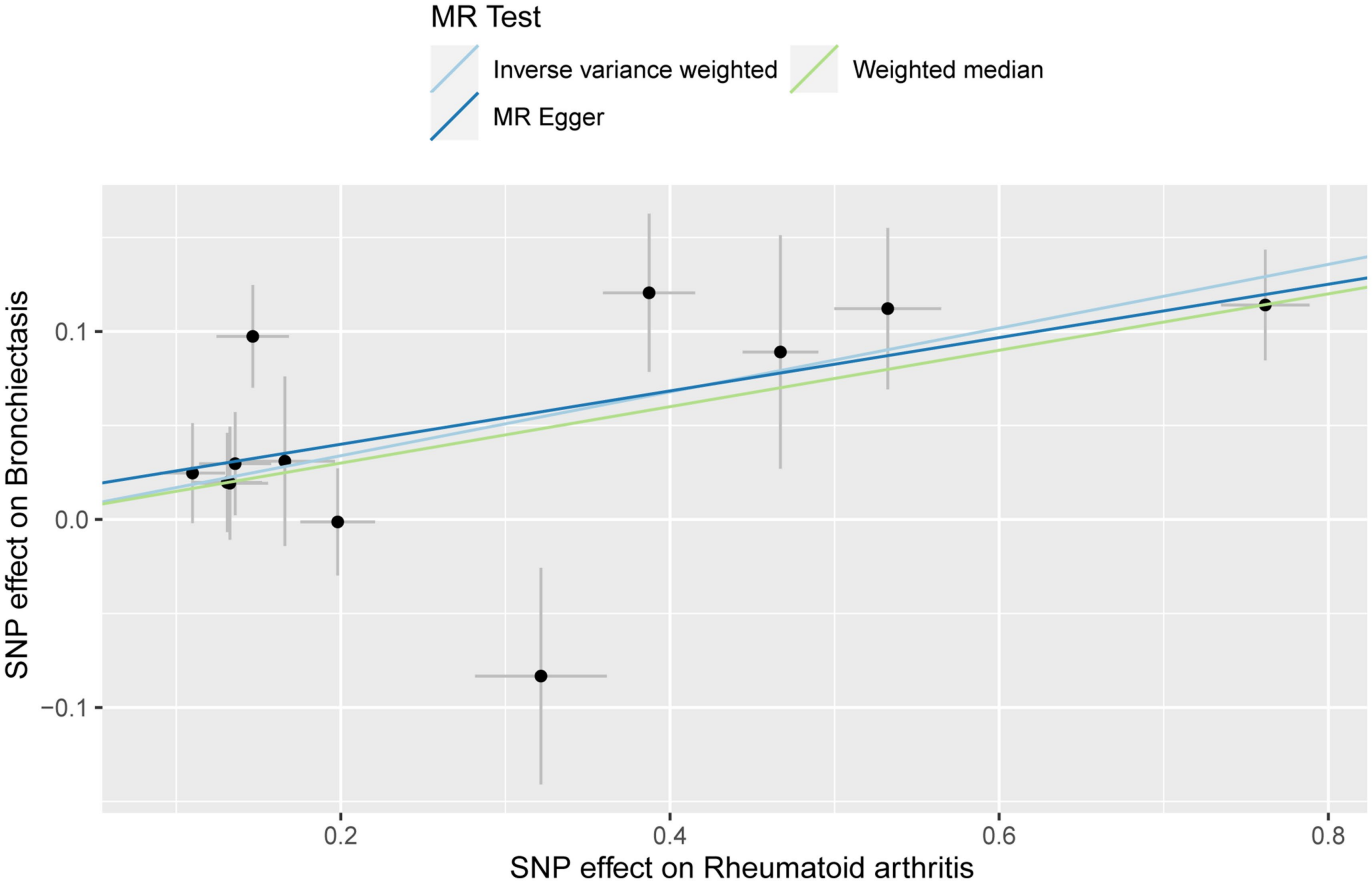
Scatter plot for the causal effect of rheumatoid arthritis on the risk of bronchiectasis.

**Figure 3:**
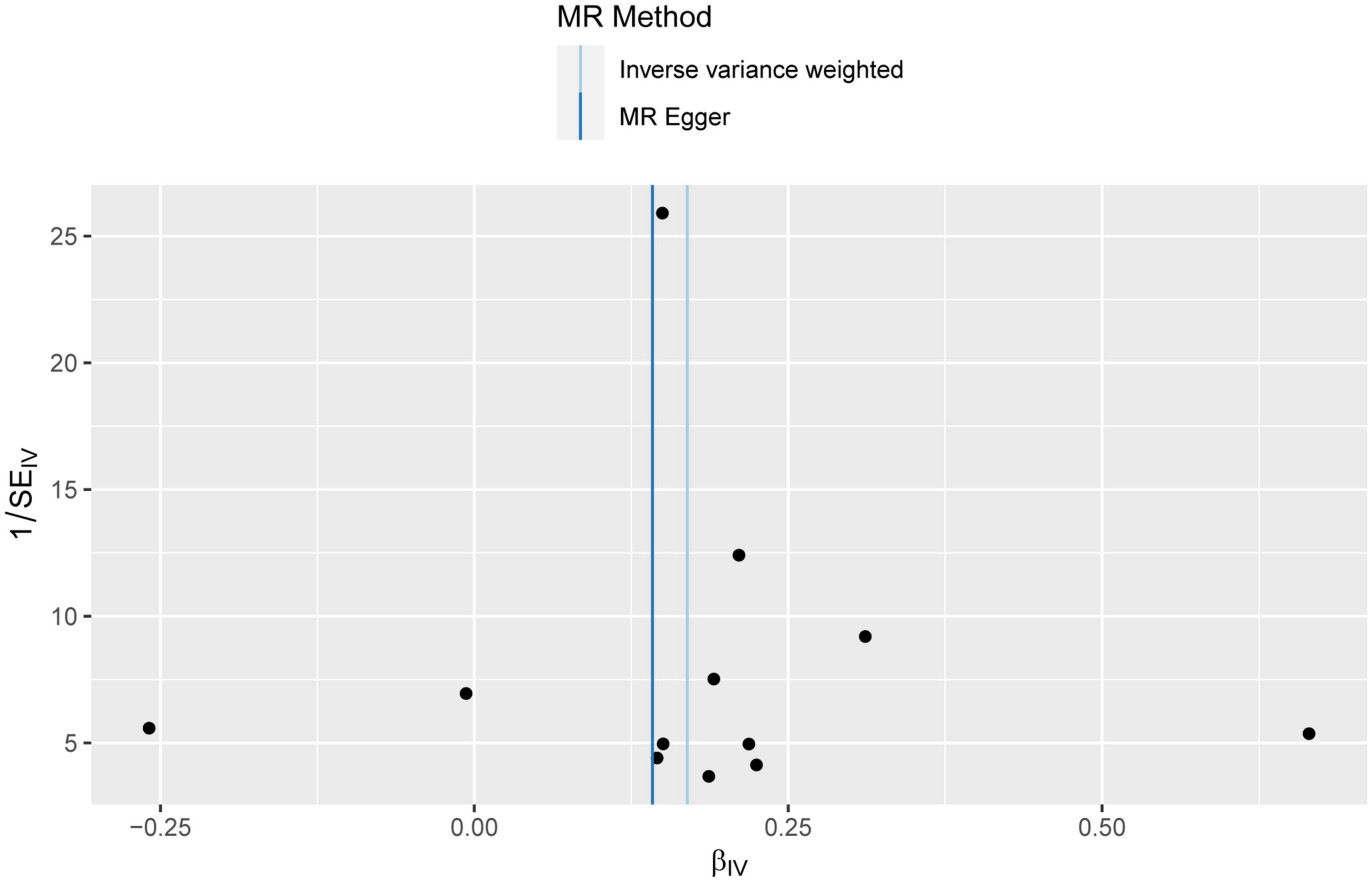
Funnel plot for the causal effect of rheumatoid arthritis on the risk of bronchiectasis.

**Figure 4:**
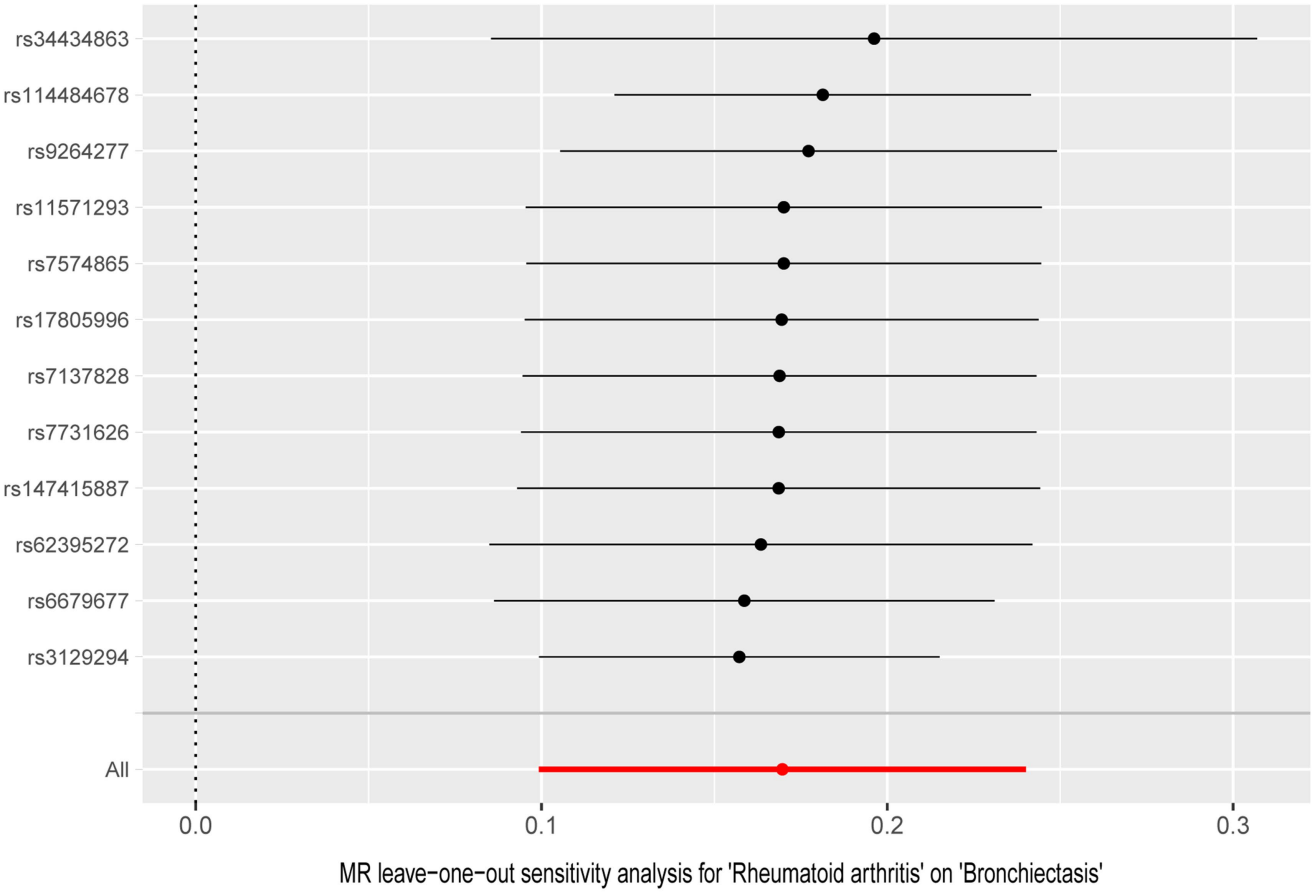
Leave-one-out analysis for the causal effect of rheumatoid arthritis on the risk of bronchiectasis.

In the UVMR analysis, 12 IVs were associated with the risk of RA following a series of quality control measures. All *F*-values for the inclusion of SNPs exceeded 10 (Supplementary Table S2). As depicted in Table 1, the primary results from the IVW method revealed a positive association between RA and the risk of bronchiectasis [odds ratio (OR) = 1.18, 95% confidence interval (CI) = 1.10–1.27, *p* = 2.34 × 10^−6^]. Meanwhile, similar risk estimates were obtained using the MR-Egger regression (OR = 1.15, 95% CI =1.03–1.29, *p* = 0.0353) and weighted median approaches (OR = 1.16, 95% CI =1.08–1.24, *p* = 2.06 × 10^−5^) (Figure 2). The Cochran Q-derived *p*-values (*p* > 0.05) suggested the absence of heterogeneity, and the MR-Egger regression intercept provided no significant evidence of horizontal pleiotropy. The funnel plot was symmetrical (Figure 3). Additionally, the LOO analysis demonstrated that the effect estimates remained unchanged despite the exclusion of any single variant (Figure 4).

**Table 1:** Causal relationships between rheumatoid arthritis (RA) and bronchiectasis risk performed by MR.

Methods	Exposure	Outcome	Method	SNPs	Beta	P	OR	95%CI	Heterogeneity	Pleiotropy
									Q_p	Egger_intercept_p
UVMR	RA	Bronchiectasis	IVW	12	0.1697	2.34 × 10^−6^	1.18	(1.10, 1.27)	0.1178	0.5515
	RA	Bronchiectasis	MR Egger	12	0.1419	0.0353	1.15	(1.03, 1.29)		
	RA	Bronchiectasis	WM	12	0.1500	2.06 × 10^−5^	1.16	(1.08, 1.24)		
Reverse MR	Bronchiectasis	RA	Wald ratio	1	0.2628	0.0562	1.30	(0.99, 1.70)	NA	NA
Replication analysis (European)	RA	Bronchiectasis	IVW	85	0.1190	0.0011	1.13	(1.05, 1.21)	0.1521	0.5444
	RA	Bronchiectasis	MR Egger	85	0.1448	0.0113	1.16	(1.04, 1.29)		
	RA	Bronchiectasis	WM	85	0.1606	0.0089	1.17	(1.04, 1.32)		
Replication analysis (EastAsian)	RA	Bronchiectasis	IVW	11	0.2655	**0.0076**	1.30	(1.07, 1.58)	0.3590	0.3204
	RA	Bronchiectasis	MR Egger	11	0.3925	0.0331	1.48	(1.09, 2.01)		
	RA	Bronchiectasis	WM	11	0.2767	0.0161	1.32	(1.05, 1.65)		

IVW, inverse variance weighted; WM, weighted median; UVMR, Univariable MR; MR, Mendelian randomization; SNPs, single nucleotide polymorphisms; NA, not available.

### Bidirectional and replication MR analysis

In the bidirectional MR analysis, there was no evidence of a causal effect of bronchiectasis on RA risk (OR = 1.30, 95% CI = 0.99–1.70, *p* = 0.0562) (Table 1). To further validate our results, we performed replication MR analysis using GWAS databases from different sources. Unsurprisingly, we observed a similar trend in European (OR = 1.13, 95% CI = 1.05–1.21, *p* = 0.0011) and East Asian populations (OR = 1.30, 95% CI = 1.07–1.58; *p* = 0.0076) (Table 1). Sensitivity analysis indicated the results were stable.

### MVMR and mediation analysis

Several factors may affect the association between RA and the risk of bronchiectasis. MVMR analysis was performed to examine whether RA has independent effects on bronchiectasis with adjustment of potential confounding factors. In the MVMR analysis, IVW estimates were consistent with the adjustments of NSAIDs (OR = 1.19, 95% CI = 1.10–1.29, *p* = 1.16 × 10^−5^), and glucocorticoids (OR = 1.18, 95% CI = 1.11–1.26, *p* = 7.34 × 10^−8^) (Table 2). However, no evidence was shown between RA and bronchiectasis after adjustment for immunosuppressants (OR = 0.99, 95% CI = 0.88–1.10, *p* = 0.7942), suggesting that immunosuppressants might play a role in the association between RA and bronchiectasis.

**Table 2:** Multivariable inverse variance weighted estimates for adjusted associations with RAs.

Exposure	Adjustment	SNPs	P	OR	95%CI
RA	NSAIDs	14	1.16 × 10^−5^	1.19	(1.10, 1.36)
RA	Glucocorticoids	33	7.34 × 10^−8^	1.18	(1.11, 1.26)
RA	Immunosuppressants	18	0.7942	0.99	(0.88, 1.10)

RA, Rheumatoid arthritis; NSAIDs, non-steroidal anti-inflammatory drugs; SNPs, single nucleotide polymorphisms; OR, odds ratio; CI, confidence interval.

We further performed mediation analyses to validate the mediating effect of immunosuppressants in bronchiectasis due to RA. The results revealed that immunosuppressants account for 58% of the association between RA and bronchiectasis (Supplementary Table S3).

## Discussion

To our knowledge, this is the first MR study to explore the causal relationship between RA and bronchiectasis. In the present study, a two-sample MR study demonstrated a causal relationship between genetically predicted RA and increased risk of bronchiectasis. Contrary to this, we found no significant association between bronchiectasis and RA. Both the MVMR and sensitivity analyses confirmed the statistical robustness of these findings.

Previous observational studies have suggested an association between RA and bronchiectasis. For example, several retrospective studies have shown that the prevalence of bronchiectasis in patients with RA ranges from 0.6 to 52.0% (31, 32), whereas the prevalence observed in prospective studies ranges from 1.9 to 58% (33, 34). The variations in prevalence rates can be largely attributed to the utilization of diverse imaging modalities, such as clinical scans, computed tomography (CT), high-resolution CT (HRCT), and additional radiological methods. In addition, several cross-sectional studies also provide some supporting evidence (34–39). In the cross-sectional study by Metafratzi et al., they found a prevalence of 58.1% bronchiectasis in early RA patients (without respiratory symptoms). Although the control group exhibited a similar frequency of bronchiectasis, the extent of the condition, as measured by HRCT scores, was substantially more severe among the RA patients compared to the controls (34). However, these study designs are limited to establishing causal inferences due to the potential for confounding bias. Randomized controlled trials (RCTs) are the gold standard for establishing causality; however, they are often limited by substantial time and financial constraints. MR can be considered a naturalistic approximation of RCTs. Therefore, our MR study provides robust evidence for a causal relationship between RA and bronchiectasis.

RA and bronchiectasis are both common diseases with a poor prognosis, and RA combined with bronchiectasis has an even worse prognosis, However, the mechanisms underlying the association of these conditions are unclear (40, 41). Current research suggests that three primary factors are likely involved in the development of the condition: inflammation, infection, and genetics. In a longitudinal population-based study by Chao et al., RA patients had a 2.12-fold increased risk of developing bronchiectasis compared with matched control patients, which remained significant after accounting for potential confounders. This elevated risk was particularly pronounced in patients with seropositive RA (SPRA), suggesting that rheumatic inflammation contributes significantly to the development of RA-bronchiectasis (8). Although NSAIDs and glucocorticoids are recognized for their proclivity to increase the risk of infections, the relationship between these medications and the heightened risk of bronchiectasis in patients with RA remains under-explored. Our MVMR analysis results suggest that these factors may not act as confounders; however, additional clinical studies are necessary to corroborate our findings. Standard RA treatments, such as corticosteroids, leflunomide, and biologic disease-modifying antirheumatic drugs (DMARDs), have been associated with an elevated risk of lower respiratory tract infections and mycobacterial lung infections, potentially leading to a marked increase in the incidence of bronchiectasis. The results of our MVMR analysis also suggest that immunosuppressants may play a role in RA leading to bronchiectasis. Mediation analyses further revealed that 58% of the effects of RA on bronchiectasis were mediated by immunosuppressants (42–44). Besides, several studies have also reported 6 genetic risk factors associated with the development of bronchiectasis, and these studies have focused on CFTR and HLA variants (45–48).

However, our study also has several limitations. First, the nature of GWAS datasets precludes the availability of comprehensive clinical details, such as the type of immunosuppressant used in RA patients, and therefore we were unable to perform subgroup analyses for different immunosuppressants. Second, due to the lack of detailed information on clinically defined or radiologically defined bronchiectasis in our GWAS data, we were unable to conduct subgroup analyses pertaining to these factors. Third, although the MR method provides us with a way of estimating causal relationships between genes and phenotypes, we cannot be completely certain of the causal relationship between RA and bronchiectasis due to the limitations of the effect size and the number of SNPs. Lastly, caution should be exercised when generalizing these findings to diverse racial or ethnic groups, given the study’s limited racial and ethnic scope.

In summary, RA could increase the risk of bronchiectasis and is mediated by immunosuppression, but there is no evidence that bronchiectasis increases the risk of RA. The findings of this study serve as a foundational point for future exploration and hold clinical relevance. Healthcare providers should be vigilant about the potential onset of bronchiectasis in patients with RA. The underlying pathophysiological mechanisms linking the development of bronchiectasis in RA patients warrant further comprehensive investigation.

## Data availability statement

The original contributions presented in the study are included in the article/Supplementary material, further inquiries can be directed to the corresponding author.

## Ethics statement

Ethical approval was not required for the studies involving humans because this study did not require separate ethical approval. The studies were conducted in accordance with the local legislation and institutional requirements. Written informed consent for participation was not required from the participants or the participants' legal guardians/next of kin in accordance with the national legislation and institutional requirements because this study did not require separate ethical approval.

## Author contributions

YL: Conceptualization, Data curation, Formal analysis, Investigation, Software, Supervision, Validation, Visualization, Writing – original draft, Writing – review & editing. WW: Data curation, Investigation, Writing – original draft. DZ: Investigation, Methodology, Writing – original draft. LL: Investigation, Methodology, Writing – original draft.

## Footnotes

^1^ https://snipa.helmholtz-muenchen.de/snipa3/index.php
