# Supplementary material for: The causal relationship between rheumatoid arthritis and bronchiectasis: a bidirectional Mendelian randomization study
Source: Front Med (Lausanne). 2024 Jun 20;11:1403851. doi: 10.3389/fmed.2024.1403851 (PMC11222563; doi:10.3389/fmed.2024.1403851)
Supplement: Supplementary file 2 [file Table_2.DOC]

| **Table S2 Harmonization data rheumatoid arthritis (RA)(FinnGen consortium) and bronchiectasis(IEU Open GWAS project) in European populations** | | | | | | | | | | | | | |
| --- | --- | --- | --- | --- | --- | --- | --- | --- | --- | --- | --- | --- | --- |
| **SNP** | **effect_allele** | **other_allele** | **beta** | **eaf** | **se** | **samplesize** | **pval** | **pos** | **chr** | **exposure** | **mr_keep** | **R2** | **F** |
| rs114484678 | C | T | 0.3216 | 0.06549 | 0.04 | 153457 | 8.81E-16 | 32215057 | 6 | RA | TRUE | 0.0004 | 64.6408 |
| rs11571293 | T | G | -0.131 | 0.3156 | 0.0211 | 153457 | 5.70E-10 | 204717713 | 2 | RA | TRUE | 0.0003 | 38.5454 |
| rs147415887 | A | AC | 0.4671 | 0.5645 | 0.023 | 153457 | 7.51E-92 | 32661745 | 6 | RA | TRUE | 0.0027 | 412.4377 |
| rs17805996 | C | T | 0.166 | 0.1223 | 0.0304 | 153457 | 4.77E-08 | 129515441 | 8 | RA | TRUE | 0.0002 | 29.8170 |
| rs2922996 | C | G | 0.2154 | 0.4031 | 0.0211 | 153457 | 1.83E-24 | 31337207 | 6 | RA | FALSE | 0.0007 | 104.2127 |
| rs3129294 | C | A | -0.1465 | 0.2815 | 0.022 | 153457 | 2.87E-11 | 33084671 | 6 | RA | TRUE | 0.0003 | 44.3429 |
| rs34434863 | G | T | 0.7617 | 0.3405 | 0.0269 | 153457 | 4.70E-176 | 32559673 | 6 | RA | TRUE | 0.0052 | 801.7846 |
| rs62395272 | T | C | 0.5323 | 0.1088 | 0.0325 | 153457 | 2.19E-60 | 31394424 | 6 | RA | TRUE | 0.0017 | 268.2505 |
| rs6679677 | A | C | 0.3873 | 0.1477 | 0.028 | 153457 | 1.27E-43 | 114303808 | 1 | RA | TRUE | 0.0012 | 191.3257 |
| rs7137828 | T | C | -0.1099 | 0.5853 | 0.0198 | 153457 | 2.62E-08 | 111932800 | 12 | RA | TRUE | 0.0002 | 30.8077 |
| rs7574865 | G | T | -0.1326 | 0.7676 | 0.0232 | 153457 | 1.04E-08 | 191964633 | 2 | RA | TRUE | 0.0002 | 32.6667 |
| rs7731626 | A | G | -0.1358 | 0.2783 | 0.0219 | 153457 | 5.51E-10 | 55444683 | 5 | RA | TRUE | 0.0003 | 38.4508 |
| rs9264277 | C | T | 0.1981 | 0.7291 | 0.0227 | 153457 | 2.60E-18 | 31224667 | 6 | RA | TRUE | 0.0005 | 76.1573 |
