# Supplementary material for: The causal relationship between rheumatoid arthritis and bronchiectasis: a bidirectional Mendelian randomization study
Source: Front Med (Lausanne). 2024 Jun 20;11:1403851. doi: 10.3389/fmed.2024.1403851 (PMC11222563; doi:10.3389/fmed.2024.1403851)
Supplement: Supplementary file 3 [file Table_3.DOC]

| **Table S3 Mediation analysis of the mediation effect between RA and bronchiectasis via immunosuppressants** | | | | | | | | |
| --- | --- | --- | --- | --- | --- | --- | --- | --- |
| exposure | outcome | method | nsnp | beta | pval | or | or_lci95 | or_uci95 |
| RA | Bronchiectasis | Inverse variance weighted | 12 | 0.1697 | 2.34478E-06 | 1.18 | 1.10 | 1.27 |
| RA | Bronchiectasis | MR Egger | 12 | 0.1419 | 0.035270137 | 1.15 | 1.03 | 1.29 |
| RA | Bronchiectasis | Weighted median | 12 | 0.1500 | 1.58129E-05 | 1.16 | 1.09 | 1.24 |
| RA | immunosuppressants | Inverse variance weighted | 13 | 0.4956 | 6.88876E-09 | 1.64 | 1.39 | 1.94 |
| RA | immunosuppressants | MR Egger | 13 | 0.4215 | 0.017460716 | 1.52 | 1.13 | 2.05 |
| RA | immunosuppressants | Weighted median | 13 | 0.5048 | 2.83263E-38 | 1.66 | 1.53 | 1.79 |
| immunosuppressants | Bronchiectasis | Inverse variance weighted | 10 | 0.1986 | 0.000254628 | 1.22 | 1.10 | 1.36 |
| immunosuppressants | Bronchiectasis | MR Egger | 10 | 0.0816 | 0.556388168 | 1.09 | 0.84 | 1.41 |
| immunosuppressants | Bronchiectasis | Weighted median | 10 | 0.2240 | 5.63742E-05 | 1.25 | 1.12 | 1.40 |
